# Case vignettes based on EQ-5D to elicit stated preferences for health services utilization from the insurees’ perspective

**DOI:** 10.1186/s12913-015-1143-2

**Published:** 2015-10-24

**Authors:** Julia Eckert, Marcel Lichters, Silke Piedmont, Bodo Vogt, Bernt-Peter Robra

**Affiliations:** Institute of Social Medicine and Health Economics, University of Magdeburg, Magdeburg, Germany; Department of Business Studies, Harz University of Applied Sciences, Wernigerode, Germany; Department of Empirical Economics, Faculty of Economics and Management, University Magdeburg, Magdeburg, Germany

**Keywords:** Health services utilization, Discrete choice, Vignettes, EQ-5D, Andersen model, Germany

## Abstract

**Background:**

There is little evidence as to why or why not insurees decide to seek medical services. Steps prior to the entry of the insuree into the professional health care system have not been sufficiently examined and can only be partially described by secondary data of the statutory health insurance (SHI). We report the first investigation using case vignettes based on the generic health-related quality of life questionnaire EQ-5D as part of a choice study to assess insurees' stated preferences in health services utilization.

**Methods:**

We invited 1500 randomly selected citizens (age 30 to 70 years) from the East German state of Saxony-Anhalt by postal mail to participate in the choice study. Attributes of the case vignettes involved in choice tasks were the five dimensions of the EQ-5D. We used multilevel mixed effects logit regression analysis with the dependent variables: preference to seek medical services (model 1) and preferred time until consultation (model 2) for the assessed case vignette. The EQ-5D attributes of the case vignettes and participant characteristics served as the independent variables. We also included the respondent's certainty of choosing from the choice set, and the order of questions of the questionnaire as control variables.

**Results:**

Of the 1500 questionnaires 683 were evaluable (net response rate 48.0 %). On the level of the case vignettes, problems in all five dimensions of the EQ-5D were statistically significant factors of the estimated likelihood to seek medical services (model 1). On the respondent level, there was a significant relationship between the preference for medical consultation for the assessed case vignette and the respondent's gender, age, educational level, the existence of a regular doctor, and the certainty of choosing from the choice set.

Problems in four of the five dimensions of the EQ-5D (except anxiety/depression) of the case vignettes were significantly associated with the preferred time until consultation (model 2). On the respondent level, gender, educational level, the certainty of choosing from the choice set, and the order of questions of the questionnaire were significant determinants of the time until consultation.

**Conclusions:**

Our study offers a promising new approach for the national and cross-national study of preferences in health services utilization from the insurees' perspective.

**Electronic supplementary material:**

The online version of this article (doi:10.1186/s12913-015-1143-2) contains supplementary material, which is available to authorized users.

## Background

The number of doctor-patient contacts in the outpatient sector in Germany is among the highest in the European Union [1]. A survey of primary care physicians conducted in eleven countries revealed similar results [2]: with a median of 250 contacts per week, doctors in Germany attended to significantly more patients than their colleagues in other countries (ranging from 50 in Sweden to 150 in Italy). With a median of 9.1 min per contact [2], it may not be surprising that patients in Germany report deficits in their doctor-patient communications [3].

While incentives from the fee schedule, as well as supply-driven demand could contribute to this pattern [4], only a few studies on demand-driven health services utilization have been conducted on the general German population [5–11], all but one [11] assessing utilization retrospectively (i.e. after the entry of insurees into the professional health care system). Moreover, they largely used secondary data of the statutory health insurance (SHI) that do not describe reasons for physician encounters well and generally lack data from insurees that do not demand medical services.

In Germany, health insurance has become mandatory for citizens and permanent residents since 2009. In 2014, around 85 % of the German population were statutorily insured, another 11 % were privately insured and the remainder belonged to special groups such as the armed forces [12]. The German health care system offers free choice of providers and unrestricted access to all care levels such as ambulatory, hospital and dental care, prescription drugs and rehabilitation regardless of the type of health insurance. Germany has one of the highest levels of health care expenditure as share of GDP in the European Union (11.2 % of GDP in 2013) [13, 14].

The aim of the present study was to construct and test a generic, i.e. not disease specific instrument to elicit stated preferences for health services utilization from the insurees' perspective. In particular, with this instrument, we wanted to shed light on the decision-making process made by insurees prior to consulting (or not) a physician.

For this purpose, the present study used discrete choice methodology to estimate the relative likelihoods of choosing utilization of health services among a set of alternatives. Discrete choice methodology has seen growing interest in health research as a method for eliciting stated preference [15–17]. Respondents are asked to choose their preferred scenario with certain characteristics (attributes) and associated specified ranges (levels) among a set of alternatives. The statistical analyses of stated preferences offers important insights about the absolute and relative contribution of alternatives' attributes in the decision process [18].

The study draws on brief, standardized health states (case vignettes) as scenarios in the present discrete choice study. Robra et al. showed that case vignettes are a useful instrument for health services research in Germany [19]. When constructing the case vignettes, we did not refer to symptom complexes that require medical knowledge but, instead, used hypothetical health states based on the five areas of the health-related quality of life questionnaire EQ-5D [20, 21].

To the best knowledge of the authors, this study is the first attempt to use case vignettes based on EQ-5D to probe insurees' stated preferences in health services utilization rather than in quality of life research. Although the information provided could ultimately inform policy decisions and thus help to formulate a more citizen-oriented health policy, this is outside the scope of the present paper. Instead, the study focuses on assessing case vignettes based on EQ-5D as a new instrument in health service utilization research.

## Methods

### Postal survey

The study team sent out a postal survey in February, 2013 to 1500 German men and women between the ages of 30 and 70 years old in the three cities Magdeburg, Wittenberg, and Stendal in the East German state of Saxony-Anhalt. Random samples were officially obtained from the respective residents’ registration offices in June, 2012. The data collection was undertaken anonymously. All addressees received a reminder letter four weeks after the first letter. The Ethics Committee of the University of Magdeburg (Germany) reviewed and approved the study (Ref 142/12).

### The EQ-5D questionnaire

The EQ-5D questionnaire is the most widely used generic instrument to measure health-related quality of life (HRQL) [22]. The first part of the EQ-5D-3L questionnaire consists of five questions with three possible answer levels ("no problems", "some or moderate problems" and "extreme problems") in the five dimensions mobility, self-care, usual activities, pain/discomfort, and anxiety/depression. A total of 243 possible health states, referred to by a five-digit code from 11111 (best HRQL) to 33333 (worst HRQL), can thus be encoded. The different five-digit health states are convertible into a single summary index by deducing weights for each of the levels in each dimension from the value for the best HRQL. In the past, value sets have been derived for various countries and regions [23].

In the second part of the questionnaire, the respondent is asked to mark his current health state on a visual analogue scale (VAS) ranging from 0 (worst imaginable health) to 100 (best imaginable health).

### Discrete choice study

The team of researchers selected the attributes and levels of the discrete choice study based on the EQ-5D-3L questionnaire. More precisely, the researchers employed the five attributes of the EQ-5D-3L with the two levels "no problems" and "some or moderate problems". Five attributes per scenario is assumed to be an acceptable number in order not to overburden the cognitive ability of the respondent [15]. We omitted the highest level "extreme problems" from our study design because we expected that participants would understand such problems as already requiring regular medical care.

Evidence shows that the acceptability to respondents is higher when realistic scenarios are chosen [24, 25]. We therefore employed the eight most common combinations as measured in the two representative population surveys conducted in Germany using the EQ-5D-3L questionnaire (ESEMeD-Study [26], W&B-Study [27]). To ensure a more balanced set of scenarios, we chose seven additional plausible combinations of functional and mental limitations, the worst possible health state being 22222. Table 1 presents the fifteen case vignettes (health states) used in our study and their respective prevalence rates within the general German population.

**Table 1:** The 15 case vignettes (health states) included in the choice study and their respective prevalence rates within the general German population

Combination of attributes and levels (health states)^a^	Prevalence in %	
	ESEMeD-Study	W&B-Study
	(*n* = 3552)	(*n* = 1966)
11111	65.9	60.6
11121	12.6	14.5
21121	5.0	3.4
21221	3.9	3.0
21111	2.8	0.9
11112	1.3	2.5
11221	1.0	2.2
11122	1.0	2.9
Total	93.5	90.0
11211	---	---
11212	---	---
12112	---	---
12211	---	---
21112	---	---
21211	---	---
22222	---	---

^a^Five dimensions of the EQ-5D: mobility, self-care, usual activities, pain/discomfort, and anxiety/depression with the two levels 1 = "no problems" and 2 = "some or moderate problems"

**Fig. 1:**
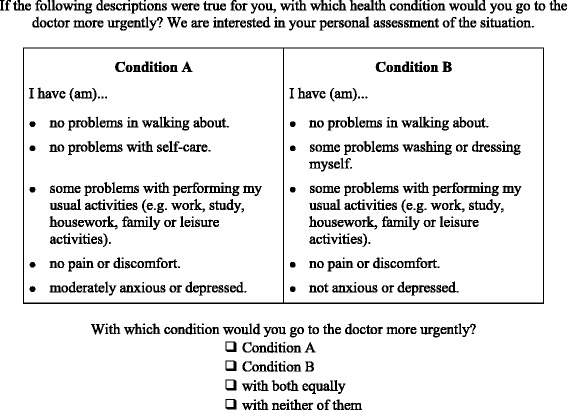
Exemplary choice task (here: condition A = 11212 vs. condition B = 12211)

Case vignettes were assigned randomly to the pairwise choice scenarios and questionnaires. Each choice task consisted of two different case vignettes. Each respondent was asked to complete four pairwise choice tasks imagining that he or she would be experiencing the health states presented. Since it is plausible for decision-makers to seek medical services with both alternatives or with neither one, we expanded the choice sets by these two choice alternatives. An exemplary choice task is shown in Fig. 1.

Furthermore, each addressee indicated the certainty of choosing from the choice set on a numeric rating scale (NRS, ranging from 1 = "very uncertain" to 10 = "very certain") and, in case of preferring to seek medical services, the preferred time until consultation ("immediately", "today", "tomorrow", "in the next weeks", "in the next months").

We randomly administered two questionnaire versions with different ordering of questions to test for possible priming effects. Priming is relevant to surveys as it activates concepts in memory that are then overly accessible in subsequent questions [28, 29]. We tested the hypothesis that priming alters preferences in health services utilization. Whereas version 1 started with questions on the respondent's own health using the EQ-5D-3L questionnaire, version 2 commenced with the presentation of pairwise choice tasks.

As a measurement of comprehension, we asked for the time needed to complete the questionnaire.

Pretesting of the questionnaire on a random sample (*n* = 90 of which 45 participated) led to minor reformulations of some questions to ensure comprehensibility and manageability.

### Andersen's Behavioral Model

Additionally, we included questions on potential individual determinants of the utilization of health services. Similar to other studies (for a review see [30, 31]), we employed Andersen's Behavioral Model as the theoretical framework to organize the multitude of potential individual determinants of the utilization of health services. This model assumes that predisposing, enabling, and need factors affect the individual's utilization of health services [32–35]. We chose characteristics from these domains as adjustors in our investigation.

We used the variables gender, age in years, and educational level as predisposing factors. As a measure of educational level, we employed the CASMIN (Comparative Analysis of Social Mobility in Industrial Nations) classification, an instrument developed to compare educational attainment internationally with the following three categories: low, middle, and high educational level [36, 37].

The employment status and whether or not the respondent had a regular doctor served as enabling factors in the present study. We included the self-reported current health state of the respondent as summarized by the EQ-5D index as a need factor. We chose the European index of the EQ-5D since it is not only based on a data pool with the largest empirical basis but also because valuations of EQ-5D health states are in considerable agreement across European countries as pointed out by Greiner et al. [38, 39].

### Statistical analysis

The statistical analyses of stated preferences was conducted by applying multilevel mixed effects logit models with random effect intercept that took into account clustering of multiple case vignettes per respondent (=4 choice tasks x 2 vignettes each). Binary dependent variables were the preference to seek medical services (yes vs. no) in the first model and the preferred time until consultation ("immediately", "today", "tomorrow" vs. "in the next weeks", "in the next months", not at all) in the second model. We grouped the possible answers "immediately", "today", and "tomorrow" into one category, while "in the next weeks", "in the next months", and not at all were grouped into another category because we conceptualized them as categories of the same gradient of health services utilization.

Most of the independent variables in the model were binary while two were continuous (age in years and the European Index of the EQ-5D). Since only 4.8 % of participants indicated a low level of education, we combined these participants with those of the middle level of education to ensure an efficient estimation of regression coefficients. We dichotomized the employment status ("employed", "in training/studying" vs. lack of employment, i.e. "retired", "unemployed/looking for work", "others") for the purpose of our analyses. The certainty of the respondent of choosing from the choice set was split at median (NRS 1–8 vs. 9–10, median = 8).

We assessed the relative importance of each case vignette’s attribute in terms of the sign, magnitude, and significance of the respective coefficient. Likewise, we analyzed participant’s and other factors (i.e. questionnaire version and certainty of choice) in terms of the sign and significance of the corresponding coefficient estimate. To ease the reading of the results, we also report odds ratios for multilevel mixed effects logit regression analyses as a different way to describe the information.

Primarily main effects were estimated. All statistical tests were two-sided and alpha was set at 0.05. We excluded all cases with missing answers from analyses involving that answer. Descriptive analyses at the respondent level were carried out using SPSS version 22.0 (SPSS Inc., Chicago, IL). Multilevel mixed effects logit regression analyses were performed using SAS version 9.3 (SAS Institute Inc., Cary, NC).

## Results

The net response rate after one postal reminder was 48.0 %. Of the 702 returned questionnaires, 683 were evaluable (12 were returned entirely blank, 7 did not have any of the four choice tasks answered).

The mean age was 54.6 years (SD = 9.7) and the percentage of female participants was 52.6 %. Participation was highest in the age group 60 to 70-years (58.4 %) and lowest among the 30 to 39-year-olds (35.7 %). The randomized allocation of the fifteen possible case vignettes on the questionnaires was consistent between the questionnaires sent out and those completed and returned. This indicated that there was no preferential bias to any of the possible case vignettes.

On average, participants completed 3.9 of the 4 choice tasks. Participants preferred health services utilization in 5.6 % for the case vignette with the best possible health state (11111) and in 74.8 % for the worst possible health state (22222). Moreover, respondents indicate that they preferred seeking medical services without delay (i.e. immediately, today or tomorrow) in 1.4 % with the best and in 26.5 % with the worst possible health state. Preferring health services utilization for the best possible health state can be interpreted as social desirability bias of minor degree.

The mean time needed to complete the questionnaire was 15.5 min (SD = 11.0). There were no statistically significant differences between the two versions regarding the time spent answering the questionnaire. Table 2 summarizes participants’ characteristics in detail.

**Table 2:** Participant characteristics (*n* = 683 subjects)

	N (%^a^)
Predisposing factors of participant	
Gender^b^	
male	318 (47)
female	359 (53)
*missing*	*6 (1)*
Age; mean (SD); *n* = 669	55 (10)
Educational level (CASMIN)^b^	
low/middle	487 (71)
high	186 (27)
*missing*	*10 (1)*
Enabling Factors of Participant	
Employment status	
employed/in training/studying	395 (58)
retired/unemployed/looking for work/others	283 (41)
*missing*	*5 (1)*
Regular doctor	
yes	652 (95)
no	27 (4)
*missing*	*4 (1)*
Need Factor of Participant	
European Index of the EQ-5D; mean (SD); *n* = 656	80 (16)
Mobility	
no problems in walking about	524 (77)
some problems in walking about	144 (21)
confined to bed	3 (0)
*missing*	12 (2)
Self-Care	
no problems with self-care	644 (94)
some problems washing or dressing oneself	19 (3)
unable to wash or dress oneself	4 (1)
*missing*	16 (2)
Usual Activities	
no problems performing usual activities	533 (78)
some problems performing usual activities	134 (20)
unable to perform usual activities	7 (1)
*missing*	9 (1)
Pain/Discomfort^b^	
no pain or discomfort	268 (39)
moderate pain or discomfort	380 (56)
extreme pain or discomfort	25 (4)
*missing*	10 (2)
Anxiety/Depression^b^	
not anxious or depressed	505 (74)
moderately anxious or depressed	156 (23)
extremely anxious or depressed	12 (2)
*missing*	10 (2)

^a^rounded to the next integer; ^b^percentages do not add up to 100 % due to rounding error; *SD* = standard deviation; *n* refers to usable data

### Preference to seek medical services

A total of 4836 valid case vignettes provided by 625 respondents created the empirical basis for our first regression model. Participants preferred to seek medical services with 1789 (37.0 %) of the case vignettes. Table 3 shows the results of the multilevel mixed effects logit regression analysis with preference to seek medical services for the assessed case vignette as the dependent variable (yes = 1).

**Table 3:** Results of the multilevel mixed effects logit regression analysis with the preference to seek medical services for the assessed case vignette as the dependent variable (625 subjects, yes = 1)

Independent Variable	Coefficient	SE	*p*-value	OR
Predisposing factors of participant				
Gender (male = 1)	0.3741	0.1269	0.0032	1.454
Age (metric in years)	−0.0156	0.0077	0.0440	0.985
Educational level (CASMIN) (high = 1)	0.2872	0.1429	0.0445	1.333
Enabling factors of participant				
Employment status (employed/in training/studying = 1)	−0.0187	0.1552	0.9042	0.982
Regular doctor (yes = 1)	0.8641	0.3260	0.0081	2.373
Need factor of participant				
European Index of the EQ-5D (metric in units)	0.0028	0.0042	0.5153	1.003
Attributes of case vignette				
Mobility (problems = 1)	0.6794	0.0777	<0.0001	1.973
Self-Care (problems = 1)	1.0295	0.0981	<0.0001	2.800
Usual Activities (problems = 1)	1.0326	0.0801	<0.0001	2.808
Pain/Discomfort (problems = 1)	1.3797	0.0786	<0.0001	3.974
Anxiety/Depression (problems = 1)	0.5137	0.0832	<0.0001	1.671
Other factors				
Version of questionnaire (first questions on own health = 1)	0.1396	0.1256	0.2665	1.150
Certainty of choice (NRS 9–10 = 1)	−0.6173	0.1019	<0.0001	0.539
Intercept	−2.6877	0.6544	<0.0001	0.068

SE = Standard Error; OR = Odds Ratio; No. observations = 4836; No. individuals = 625; −2 Res Log Pseudo-Likelihood = 22,676.14; Pseudo-AIC = 22,678.14; Pseudo-BIC = 22,682.58. The difference of the study sample (*n* = 683) to 625 subjects in the regression analysis is due to missing values in the dependent and/or independent variables

On the vignette level, problems in all of the five dimensions of the EQ-5D showed a plausible significant positive association with the likelihood to seek medical services for the assessed case vignette (*p* < 0.0001 each). Problems in the vignette attribute pain/discomfort had the highest (coefficient = 1.3797) and in the attribute anxiety/depression (coefficient = 0.5137) the lowest relative importance.

On the individual level, we observed a significant positive relationship between the preference to consult a doctor and male gender, a high educational level, and having a regular doctor (*p* < 0.05 each). On the contrary, age showed a significant negative association with the preference to seek medical services (*p* < 0.05). The employment status, as well as the self-reported current health state of the respondent (the European index of the EQ-5D), were not significantly associated with the likelihood to seek medical services.

In the first regression model, we could not confirm our priming hypothesis: whether or not answering questions related to their own health using the EQ-5D beforehand did not significantly influence the preference to seek medical services for the assessed case vignette. Yet, respondents who were not very certain of their choice demonstrated a significantly higher preference for seeking medical services (*p* < 0.0001).

### Preferred time until consultation

Of the 4786 case vignettes provided by 624 subjects, 475 (9.9 %) prompted respondents to indicate that they preferred to go to the doctor without delay (i.e. immediately, today or tomorrow = 1), while 4311 (90.1 %) caused respondents to indicate that they preferred to go to the doctor in the next weeks, months, or not at all (=0). Table 4 presents the results of the binary multilevel mixed effects logit regression analysis with the preferred time until consultation as the dependent variable.

**Table 4:** Results of the multilevel mixed effects logit regression analysis with the preferred time until consultation for the assessed case vignette as the dependent variable (624 subjects, immediately, today or tomorrow = 1)

Independent Variable	Coefficient	SE	*p*-value	OR
Predisposing factors of participant				
Gender (male = 1)	0.5320	0.2129	0.0125	1.702
Age (metric in years)	−0.0003	0.0133	0.9797	1.000
Educational level (CASMIN) (high = 1)	−0.5839	0.2530	0.0211	0.558
Enabling factors of participant				
Employment status (employed/in training/studying = 1)	0.1874	0.2665	0.4820	1.206
Regular doctor (yes = 1)	0.5984	0.5912	0.3115	1.819
Need factor of participant				
European Index of the EQ-5D (metric in units)	0.0031	0.0071	0.6560	1.003
Attributes of case vignette				
Mobility (problems = 1)	0.4390	0.1315	0.0009	1.551
Self-Care (problems = 1)	0.9993	0.1609	<0.0001	2.716
Usual Activities (problems = 1)	0.6914	0.1415	<0.0001	1.996
Pain/Discomfort (problems = 1)	1.0842	0.1313	<0.0001	2.957
Anxiety/Depression (problems = 1)	0.1489	0.1455	0.3061	1.161
Other factors				
Version of questionnaire (first questions on own health = 1)	0.8070	0.2127	0.0002	2.241
Certainty of choice (NRS 9–10 = 1)	0.8070	0.1764	<0.0001	2.241
Intercept	−6.2369	1.1437	<0.0001	0.002

SE = Standard Error; OR = Odds Ratio; No. observations = 4786; No. individuals = 624; −2 Res Log Pseudo-Likelihood = 27,847.95; Pseudo-AIC = 27,849.95; Pseudo-BIC = 27,854.39. The difference of the study sample (*n* = 683) to 624 subjects in the regression analysis is due to missing values in the dependent and/or independent variables

In the analysis taking into account multiple vignettes assessed by the same respondent, problems in four of the five dimensions of the EQ-5D: mobility, self-care, usual activities, and pain/discomfort were significantly associated with the preferred time until consultation (*p* < 0.001 each). The dimension pain/discomfort showed the strongest relative relationship with the dependent variable (coefficient = 1.0842), whereas the dimension anxiety/depression showed no significant association with the time until consultation (*p* = 0.3061).

Male participants and those with a low or middle educational level were significantly more likely to state that they preferred seeking medical services without delay (*p* < 0.05 each). All other examined participant factors showed—ceteris paribus—no significant association with the time until consultation.

This model unveils a priming effect for the time until consultation: respondents who had reflected upon their own health before completing the choice tasks had a higher likelihood to prefer consulting a doctor without delay (*p* < 0.001). Moreover, participants that were very certain of choosing from the choice set also had a higher probability to state that they preferred seeking medical services without delay (*p* < 0.0001).

### Further analyses^1^

We performed additional analyses to further test for robustness of the two estimated models. First, we ran both models without the variables “version of questionnaire” and “certainty of choice” to test for possible confounding effects on the other factors. The signs of the independent variables and the relative importance of each attribute of the case vignette remained unchanged in the reduced compared to the full model. Furthermore, in the reduced models, only the variable educational level in model 1 became non-significant (p-value changed from 0.0445 to 0.0618), with the significance of all the other predictors remaining unchanged (alpha set at 0.05). Comparing log pseudo-likelihoods shows that adding these two variables to our model results in a statistically significant improvement in model fit (*p* < 0.01). Thus, we decided to control for the questionnaire version and certainty of choice since their inclusion not only improves model fit but the authors also consider it important to ensure construct validity of the vignette approach.

Second, to further test for the importance of age, we also ran the same models with age as a categorical variable with the four age bands 30 to 39, 40 to 49, 50 to 59 and 60 to 70 years. Having age as a categorical variable did not alter the signs of the other independent variables in both models. With the exception of educational level in model 1 where p-value changed from 0.0445 to 0.0581, the significance of the other independent variables also remained unchanged (alpha set at 0.05).

Third, to shed even more light into the age effect, we computed the three interactions age x gender, age x gender x education, age x gender x employment. None of these interactions was significant in the first model. As for the preferred time until consultation, male participants of advanced age had a higher likelihood to seek medical services without delay (coefficient = 0.0477, *p* = 0.0356). Furthermore, male participants of advanced age with a low or medium level of education were also more likely to consult a doctor without delay (coefficient = 0.0538, *p* = 0.0189). The interaction age x gender x employment showed no significant relationship with the preferred time until consultation.

Lastly, with regard to the priming effect detected for the preferred time until consultation, we applied the second regression model separately for each questionnaire version (although without the variable "regular doctor" as a consequence of too few respondents with no regular doctor in the two separate versions). This did not alter the signs of the significant coefficients, providing further support for the robustness of the obtained results.

## Discussion

The present research is the first to elicit stated preferences of seeking medical services from the insurees’ perspective through a discrete choice approach involving case vignettes based on EQ-5D. The results suggest that the chosen approach seems to be feasible to examine preferences for health services utilization.

On the vignette level, results showed plausible significant positive relationships of moderate health problems with the preference to seek medical services for the assessed case vignette. As for the preferred time until consultation, problems in all five dimensions of the EQ-5D also had plausible positive signs and four of the five attributes of the case vignette were statistically significant. Consequently, the presented results of the discrete choice experiment are useful for estimating the contribution of hypothetical moderate problems within the five EQ-5D areas in the respondents' decision of choosing health services utilization.

Participants stated they are more likely to choose utilization of health services for moderate pain or discomfort (coefficient = 1.3797), some problems washing or dressing and some problems with performing usual activities (coefficient = 1.0295 and 1.0326 respectively) than with some problems in walking about (coefficient = 0.6794) or being moderately anxious or depressed (coefficient = 0.5137).

Similarly, the likelihood to seek health services without delay (i.e. immediately, today or tomorrow) was also higher for moderate pain or discomfort (coefficient = 1.0842), some problems with washing or dressing (coefficient = 0.9993) than with some problems performing usual activities (coefficient = 0.6914) or some problems in walking about (coefficient = 0.4390). Being moderately anxious or depressed showed no significant relationship with the preferred time until consultation (*p* = 0.3061).

Pain, and especially chronic pain, is a serious health problem with significant consequences for the quality of life for those affected and a frequent reason of medical encounter [40, 41]. This notion is compatible with our findings. Moderate pain and discomfort had the highest prevalence rate among our participants (55.6 %). In the present study, pain and discomfort also had the highest relative importance for both the preference to seek medical services and the preferred time until consultation.

Moderate problems in the EQ-5D dimension anxiety and depression had the second highest prevalence rate among our study population (22.8 %). Although it remains difficult to determine the exact magnitude of mental disorders, according to claims data, depressive disorders show lower prevalence rates in the Eastern than in the Western part of Germany [42]. Apparently, many affected persons do not actively seek help in the regions of our study. This might be in fear of discrimination and stigmatization [43, 44]. Likewise, respondents might not trust in doctor's ability to effectively treat anxiety or depressive disorders. Despite their high prevalence among this study’s participants, anxiety and depression had the lowest relative importance on the preference to seek medical services and showed no significant relationship with the preferred time until consultation in the discrete choice experiment. There is a need for health literacy about anxiety and depressive disorders in order to reduce the differential in stated preference.

Moderate problems with self-care had the lowest prevalence rate among participants in the present study (2.8 %). However, problems with washing or dressing had a high relative likelihood of health services utilization preference in the discrete choice experiment. It is worth pointing out that in the European single summary index of the EQ-5D, moderate problems with self-care have the highest aggregated coefficient among all moderate problems in the EQ-5D dimensions, i.e. a high impact on the perceived individual health state [38].

Although some problems performing usual activities have the lowest aggregated coefficient in the European single summary index of the EQ-5D, participants in the present discrete choice experiment stated they are more likely to choose utilization of health services for some problems with performing their usual activities than with e.g. some problems with mobility.

Problems walking about showed the second lowest likelihood to prefer health services utilization and the lowest significant likelihood to prefer seeking medical services without delay in the discrete choice experiment. Some problems with mobility also had the second lowest aggregation coefficient (after some problems with performing usual activities) in the European single summary index of the EQ-5D, i.e. have a relatively low impact on the perceived health state of the individual.

At the respondent level, gender was a significant predictor of the preference to seek medical services. Male participants had a higher likelihood to demand medical services for the assessed case vignette. This finding apparently contradicts the results of other studies [5, 7–9]. However, we employed case vignettes based on the generic EQ-5D questionnaire in our study and did not refer to any gender-specific symptoms. According to Rattay et al. [10], gender differences in health services utilization are especially accentuated during childbearing years but lessen or even vanish with age . Moreover, in a previous case vignette study on health services utilization, men tended to rate case vignettes as more severe than women [11]. In our study, men were also more likely to prefer seeking medical services without delay.

Younger respondents had a higher likelihood than older ones to prefer seeking medical services in our study after controlling for all other factors. However, age was not a significant predictor of the preferred time until consultation. Retrospective studies show an overall positive relationship between age and health services utilization [5, 6, 9]. According to an analysis of synergetic effects between age and the variables gender, education and employment, male participants of advanced age in general and male participants of advanced age with a low or medium level of education had a higher likelihood to prefer seeking medical services without delay compared to other participants in our study.

Respondents with a high educational level showed a higher tendency to prefer seeking medical services for the assessed case vignettes than those with lower levels of education. Still, the effect of social class including education on health services utilization has been shown to differ between consultations at specialists and primary care physicians [7, 10]. Although highly educated respondents were more likely to prefer seeking medical services for the assessed vignettes, respondents with a low or middle educational level would more likely do so without delay. This may be an opportunity to implement health literacy programs to narrow the gap between stated preferences of the social strata.

Regarding employment status, we expected employed persons to less likely prefer consulting a doctor for the evaluated case vignettes compared to respondents without employment similar to findings by Thode et al. [7]. That study assumed employed persons to have a more restricted time budget and poorer accessibility of health care facilities. However, the employment status in our study was neither a significant predictor of the preference to seek medical services nor of the preferred time until consultation holding all other independent variables fixed in the model.

The existence of a regular doctor showed a significant positive relationship with the preference to seek medical services for the assessed case vignettes. Whether having a regular doctor is a determinant or consequence of health services utilization remains unclear [45] and cannot be concluded from our data. Having a regular doctor or not showed, however, no significant relationship with the preferred time until consultation.

In this study, we expected a relationship between the self-reported current health state as summarized by the European index of the EQ-5D and the stated preference for health services utilization to the extent that respondents are unable to be completely objective and ignore their own health situation. Yet, when controlling for the other independent variables on the vignette and respondent levels, we could not detect a significant relationship of the respondent's EQ-5D index value with neither the preference to seek medical services nor the preferred time until consultation. The same was true for the EQ-5D VAS of the respondent (data not shown). This indicates that participants were sufficiently able to imagine being in the health states of the presented case vignettes while making their decisions. This supports the internal validity of our findings.

An important effect on the likelihood to seek medical services was the participant's certainty of choosing from the choice set. Respondents who were not very certain of their choice (NRS 1–8) were more likely to prefer seeking medical care for the assessed case vignettes, prompting the adage: "When in doubt, consult the doctor". Yet, only those respondents that were very certain of their choices (NRS 9–10) were significantly more likely to prefer seeking medical services without delay, i.e. immediately, today or tomorrow.

We were not able to confirm our hypothesis that priming alters preferences in seeking medical services for the assessed case vignette. However, we detected a priming effect for the preferred time until consultation: respondents who had first answered questions regarding their own health state were significantly more likely to prefer consulting a doctor without a delay. This suggests that priming influences — at least for the time until consultation — the preferences of respondents in our study. To the best of our knowledge, no one has thus far discussed the concept of priming in the context of health-related discrete choice studies. Due to its immense importance on the objectivity, reproducibility, and testability of discrete choice experiments, future work is urgently required in this area.

### Limitations

We acknowledge several limitations of this study. As participation in the study was anonymous and on a voluntary basis, selective non-response cannot be ruled out. Indeed, the response rate was lower in the younger age brackets than in the older age groups. In addition, we invited only citizens between 30 and 70 years from the East German state of Saxony-Anhalt to participate.

Although mean VAS values of our study population compare well with representative population surveys conducted in Germany [26, 27] (data not shown), future studies testing the external validity of our results on both the national and cross-national level are needed.

Furthermore, we chose solely plausible scenarios for the present discrete choice study. By reducing the number of admissible vignettes, we compromised on orthogonality and attribute balance of the choice design. Yet, we regarded the design matrix as acceptable as the maximum empirical Pearson correlation coefficient between two attributes is 0.265 (between the EQ-5D dimensions self-care and anxiety/depression).

It is obvious that future studies would benefit from the inclusion of other individual characteristics (e.g. marital status, ethnicity, income). Including health care system variables such as the rate of health insurance coverage, the density of physicians, and population health indices may also be interesting, especially for cross-national comparison. Due to the cross-sectional design, conclusions about causality of the relationships studied can only be inferred. Longitudinal studies may help to elucidate the inherent dynamics of contextual and individual factors behind health services utilization. Since we primarily estimated main effects, future studies should investigate further interactions of independent variables, which might also be considered in the study design and a priori sample size calculation. The comparative analysis of stated and observed health services utilization is a desideratum and fruitful subject of future studies.

## Conclusions

The discrete choice approach seems to be a feasible method to study health services utilization preferences from the insurees' perspective. We were able to validate the tendency to seek medical services through case vignettes based on the generic health-related quality of life questionnaire EQ-5D. Case vignettes based on EQ-5D provide a practical instrument for health services utilization research. Not only did attributes of the case vignette (the health state) contribute to the preference for seeking medical services but also characteristics of the respondent. Although future research is indicated to confirm our results in national and cross-national studies, our study offers a promising new approach for health services utilization research.

## Additional file

Additional file 1:
**Supplementary analyses.** (DOCX 67 kb)

## Footnotes

1 The authors would like to thank the reviewers for valuable suggestions regarding further analyses. See Additional file 1: Online Appendix for the results.
