# Supplementary material for: Case vignettes based on EQ-5D to elicit stated preferences for health services utilization from the insurees’ perspective
Source: BMC Health Serv Res. 2015 Oct 24;15:481. doi: 10.1186/s12913-015-1143-2 (PMC4619295; doi:10.1186/s12913-015-1143-2)
Supplement: Additional file 1: — Supplementary analyses. (DOCX 67 kb) [file 12913_2015_1143_MOESM1_ESM.docx]

## Table 1 - Results of the multilevel mixed effects logit regression analysis with the preference to seek medical services for the assessed case vignette as the dependent variable (630 subjects, yes=1, without the variables "version of questionnaire" and "certainty of choice")

| **Independent Variable** | **Coefficient** | **SE** | **p-value** | **OR** |
| --- | --- | --- | --- | --- |
| **Predisposing factors of participant** |  |  |  |  |
| Gender (male = 1) | 0.4137 | 0.1261 | 0.0010 | 1.512 |
| Age (metric in years) | -0.0162 | 0.0077 | 0.0351 | 0.984 |
| Educational level (CASMIN) (high = 1) | 0.2654 | 0.1421 | 0.0618 | 1.304 |
| **Enabling factors of participant** |  |  |  |  |
| Employment status (employed/in training/studying = 1) | -0.0281 | 0.1543 | 0.8553 | 0.972 |
| Regular doctor (yes = 1) | 0.8666 | 0.3242 | 0.0075 | 2.379 |
| **Need factor of participant** |  |  |  |  |
| European Index of the EQ-5D (metric in units) | 0.0017 | 0.0042 | 0.6902 | 1.002 |
| **Attributes of case vignette** |  |  |  |  |
| Mobility (problems = 1) | 0.6715 | 0.0766 | <0.0001 | 1.957 |
| Self-Care (problems = 1) | 1.0152 | 0.0971 | <0.0001 | 2.760 |
| Usual Activities (problems = 1) | 1.0223 | 0.0791 | <0.0001 | 2.779 |
| Pain/Discomfort (problems = 1) | 1.3706 | 0.0774 | <0.0001 | 3.938 |
| Anxiety/Depression (problems = 1) | 0.5118 | 0.0821 | <0.0001 | 1.668 |
| **Intercept** | -2.7807 | 0.6463 | <0.0001 | 0.062 |

SE=Standard Error; OR = Odds Ratio; No. observations = 4948; No. individuals = 630; -2 Res Log Pseudo-Likelihood = 23,174.66; Pseudo-AIC = 23,176.66; Pseudo-BIC = 23,181.11. The difference of the study sample (n=683) to 630 subjects in the regression analysis is due to missing values in the independent and/or dependent variables.

## Table 2 - Results of the multilevel mixed effects logit regression analysis with the preferred time until consultation for the assessed case vignette as the dependent variable (629 subjects, immediately, today or tomorrow=1, without the variables "version of questionnaire" and "certainty of choice")

| **Independent Variable** | **Coefficient** | **SE** | **p-value** | **OR** |
| --- | --- | --- | --- | --- |
| **Predisposing factors of participant** |  |  |  |  |
| Gender (male = 1) | 0.5105 | 0.2127 | 0.0165 | 1.666 |
| Age (metric in years) | -0.0009 | 0.0133 | 0.9453 | 0.999 |
| Educational level (CASMIN) (high = 1) | -0.5013 | 0.2521 | 0.0468 | 0.606 |
| **Enabling factors of participant** |  |  |  |  |
| Employment status (employed/in training/studying = 1) | 0.1685 | 0.2678 | 0.5292 | 1.184 |
| Regular doctor (yes = 1) | 0.6138 | 0.5823 | 0.2919 | 1.847 |
| **Need factor of participant** |  |  |  |  |
| European Index of the EQ-5D (metric in units) | 0.0043 | 0.0070 | 0.5418 | 1.004 |
| **Attributes of case vignette** |  |  |  |  |
| Mobility (problems = 1) | 0.4390 | 0.1296 | 0.0007 | 1.551 |
| Self-Care (problems = 1) | 0.9789 | 0.1591 | <0.0001 | 2.661 |
| Usual Activities (problems = 1) | 0.6519 | 0.1394 | <0.0001 | 1.919 |
| Pain/Discomfort (problems = 1) | 1.1088 | 0.1296 | <0.0001 | 3.031 |
| Anxiety/Depression (problems = 1) | 0.1514 | 0.1435 | 0.2915 | 1.163 |
| **Intercept** | -5.4925 | 1.1303 | <0.0001 | 0.004 |

SE=Standard Error; OR = Odds Ratio; No. observations = 4897; No. individuals = 629; -2 Res Log Pseudo-Likelihood = 28,228.69; Pseudo-AIC = 28,230.69; Pseudo-BIC = 28,235.13. The difference of the study sample (n=683) to 629 subjects in the regression analysis is due to missing values in the independent and/or dependent variables.

## Table 3 - Results of the multilevel mixed effects logit regression analysis with the preference to seek medical services for the assessed case vignette as the dependent variable (625 subjects, yes=1, age categorial in years)

| **Independent Variable** | **Coefficient** | **SE** | **p-value** | **OR** |
| --- | --- | --- | --- | --- |
| **Predisposing factors of participant** |  |  |  |  |
| Gender (male = 1) | 0.3902 | 0.1275 | 0.0022 | 1.477 |
| Age (categorial in years); F = 1.95, p = 0.1194  30-39  40-49  50-59  60-70 | 0.6259  0.2038  0.2305  Ref | 0.2602  0.1998  0.1781 | 0.0162  0.3078  0.1955 | 1.870  1.226  1.259 |
| Educational level (CASMIN) (high = 1) | 0.2739 | 0.1445 | 0.0581 | 1.315 |
| **Enabling factors of participant** |  |  |  |  |
| Employment status (employed/in training/studying = 1) | -0.0176 | 0.1639 | 0.9145 | 0.983 |
| Regular doctor (yes = 1) | 0.8672 | 0.3265 | 0.0079 | 2.380 |
| **Need factor of participant** |  |  |  |  |
| European Index of the EQ-5D (metric in units) | 0.0026 | 0.0042 | 0.5358 | 1.003 |
| **Attributes of case vignette** |  |  |  |  |
| Mobility (problems = 1) | 0.6793 | 0.0777 | <0.0001 | 1.972 |
| Self-Care (problems = 1) | 1.0297 | 0.0982 | <0.0001 | 2.800 |
| Usual Activities (problems = 1) | 1.0346 | 0.0802 | <0.0001 | 2.814 |
| Pain/Discomfort (problems = 1) | 1.3808 | 0.0786 | <0.0001 | 3.978 |
| Anxiety/Depression (problems = 1) | 0.5131 | 0.0833 | <0.0001 | 1.671 |
| **Other factors** |  |  |  |  |
| Version of questionnaire (first questions on own health = 1) | 0.1305 | 0.1260 | 0.3005 | 1.139 |
| Certainty of choice (NRS 9-10 = 1) | -0.6065 | 0.1022 | <0.0001 | 0.545 |
| **Intercept** | -3.7127 | 0.4905 | <0.0001 | 0.024 |

SE=Standard Error; OR = Odds Ratio; Ref = Reference; F = Type III F-statistic; p = p-value; No. observations = 4836; No. individuals = 625; -2 Res Log Pseudo-Likelihood = 22,683.06; Pseudo-AIC = 22,685.06; Pseudo-BIC = 22,689.50. The difference of the study sample (n=683) to 625 subjects in the regression analysis is due to missing values in the independent and/or dependent variables.

## Table 4 - Results of the multilevel mixed effects logit regression analysis with the preferred time until consultation for the assessed case vignette as the dependent variable (624 subjects, immediately, today or tomorrow=1, age categorial in years)

| **Independent Variable** | **Coefficient** | **SE** | **p-value** | **OR** |
| --- | --- | --- | --- | --- |
| **Predisposing factors of participant** |  |  |  |  |
| Gender (male = 1) | 0.5203 | 0.2138 | 0.0150 | 1.683 |
| Age (categorial in years); F = 1.04; p = 0.3731  30-39  40-49  50-59  60-70 | -0.2293  0.3720  0.3392 | 0.4681  0.3348  0.2997 | 0.6243  0.2666  0.2578 | 0.795  1.451  1.404 |
| Educational level (CASMIN) (high = 1) | -0.5423 | 0.2562 | 0.0343 | 0.581 |
| **Enabling factors of participant** |  |  |  |  |
| Employment status (employed/in training/studying = 1) | 0.0322 | 0.2787 | 0.9081 | 1.033 |
| Regular doctor (yes = 1) | 0.5804 | 0.5964 | 0.3305 | 1.787 |
| **Need factor of participant** |  |  |  |  |
| European Index of the EQ-5D (metric in units) | 0.0038 | 0.0071 | 0.5921 | 1.004 |
| **Attributes of case vignette** |  |  |  |  |
| Mobility (problems = 1) | 0.4427 | 0.1316 | 0.0008 | 1.557 |
| Self-Care (problems = 1) | 1.0031 | 0.1611 | <0.0001 | 2.727 |
| Usual Activities (problems = 1) | 0.6960 | 0.1417 | <0.0001 | 2.006 |
| Pain/Discomfort (problems = 1) | 1.0895 | 0.1314 | <0.0001 | 2.973 |
| Anxiety/Depression (problems = 1) | 0.1570 | 0.1457 | 0.2816 | 1.170 |
| **Other factors** |  |  |  |  |
| Version of questionnaire (first questions on own health = 1) | 0.8039 | 0.2134 | 0.0002 | 2.234 |
| Certainty of choice (NRS 9-10 = 1) | 0.7911 | 0.1767 | <0.0001 | 2.206 |
| **Intercept** | -6.3919 | 0.8635 | <0.0001 | 0.002 |

SE=Standard Error; OR = Odds Ratio; Ref = Reference; F = Type III F-statistic; p = p-value; No. observations = 4786; No. individuals = 624; -2 Res Log Pseudo-Likelihood = 27,895.02; Pseudo-AIC = 27,897.02; Pseudo-BIC = 27,901.46. The difference of the study sample (n=683) to 624 subjects in the regression analysis is due to missing values in the independent and/or dependent variables.

## Table 5 - Results of the multilevel mixed effects logit regression analysis with the preference to seek medical services for the assessed case vignette as the dependent variable (625 subjects, yes=1, interaction age x gender)

| **Independent Variable** | **Coefficient** | **SE** | **p-value** | **OR** |
| --- | --- | --- | --- | --- |
| **Predisposing factors of participant** |  |  |  |  |
| Gender (male = 1) | 0.2930 | 0.7263 | 0.6867 | 1.340 |
| Age (metric in years) | -0.0162 | 0.0096 | 0.0918 | 0.984 |
| Educational level (CASMIN) (high = 1) | 0.2860 | 0.1436 | 0.0464 | 1.331 |
| **Enabling factors of participant** |  |  |  |  |
| Employment status (employed/in training/studying = 1) | -0.0177 | 0.1557 | 0.9094 | 0.982 |
| Regular doctor (yes = 1) | 0.8613 | 0.3276 | 0.0086 | 2.366 |
| **Need factor of participant** |  |  |  |  |
| European Index of the EQ-5D (metric in units) | 0.0027 | 0.0043 | 0.5248 | 1.003 |
| **Attributes of case vignette** |  |  |  |  |
| Mobility (problems = 1) | 0.6797 | 0.0777 | <0.0001 | 1.973 |
| Self-Care (problems = 1) | 1.0300 | 0.0982 | <0.0001 | 2.801 |
| Usual Activities (problems = 1) | 1.0330 | 0.0801 | <0.0001 | 2.810 |
| Pain/Discomfort (problems = 1) | 1.3806 | 0.0786 | <0.0001 | 3.977 |
| Anxiety/Depression (problems = 1) | 0.5140 | 0.0833 | <0.0001 | 1.672 |
| **Other factors** |  |  |  |  |
| Version of questionnaire (first questions on own health = 1) | 0.1393 | 0.1258 | 0.2684 | 1.149 |
| Certainty of choice (NRS 9-10 = 1) | -0.6174 | 0.1020 | <0.0001 | 0.539 |
| **Interactions** |  |  |  |  |
| Age x gender; F = 0.01, p = 0.9095 |  |  |  |  |
| Age x male | 0.0015 | 0.0132 | 0.9095 | 1.001 |
| Age x female | Ref |  |  |  |
| **Intercept** | -2.6469 | 0.7525 | 0.0005 | 0.071 |

SE=Standard Error; OR = Odds Ratio; Ref = Reference; F = Type III F-statistic; p = p-value; No. observations = 4836; No. individuals = 625; -2 Res Log Pseudo-Likelihood = 22,687.57; Pseudo-AIC = 22,689.57; Pseudo-BIC = 22,694.01. The difference of the study sample (n=683) to 625 subjects in the regression analysis is due to missing values in the independent and/or dependent variables.

## Table 6 - Results of the multilevel mixed effects logit regression analysis with the preference to seek medical services for the assessed case vignette as the dependent variable (625 subjects, yes=1, interaction age x gender x educational level)

| **Independent Variable** | **Coefficient** | **SE** | **p-value** | **OR** |
| --- | --- | --- | --- | --- |
| **Predisposing factors of participant** |  |  |  |  |
| Gender (male = 1) | 0.3151 | 0.7299 | 0.6660 | 1.370 |
| Age (metric in years) | -0.0149 | 0.0104 | 0.1509 | 0.985 |
| Educational level (CASMIN) (high = 1) | 0.4998 | 0.7992 | 0.5317 | 1.648 |
| **Enabling factors of participant** |  |  |  |  |
| Employment status (employed/in training/studying = 1) | -0.0167 | 0.1561 | 0.9146 | 0.983 |
| Regular doctor (yes = 1) | 0.8964 | 0.3305 | 0.0067 | 2.451 |
| **Need factor of participant** |  |  |  |  |
| European Index of the EQ-5D (metric in units) | 0.0026 | 0.0043 | 0.5375 | 1.003 |
| **Attributes of case vignette** |  |  |  |  |
| Mobility (problems = 1) | 0.6792 | 0.0778 | <0.0001 | 1.972 |
| Self-Care (problems = 1) | 1.0310 | 0.0982 | <0.0001 | 2.804 |
| Usual Activities (problems = 1) | 1.0332 | 0.0802 | <0.0001 | 2.810 |
| Pain/Discomfort (problems = 1) | 1.3817 | 0.0787 | <0.0001 | 3.982 |
| Anxiety/Depression (problems = 1) | 0.5144 | 0.0833 | <0.0001 | 1.673 |
| **Other factors** |  |  |  |  |
| Version of questionnaire (first questions on own health = 1) | 0.1293 | 0.1266 | 0.3069 | 1.138 |
| Certainty of choice (NRS 9-10 = 1) | -0.6201 | 0.1022 | <0.0001 | 0.538 |
| **Interactions** |  |  |  |  |
| Age x gender x educational level (CASMIN); F = 0.30, p = 0.8221 |  |  |  |  |
| Age x male x CASMIN = 3 | -0.0017 | 0.0188 | 0.9265 | 0.998 |
| Age x male x CASMIN = 1-2 | -0.0002 | 0.0134 | 0.9884 | 1.000 |
| Age x female x CASMIN = 3 | -0.0062 | 0.0147 | 0.6717 | 0.994 |
| Age x female x CASMIN = 1-2 | Ref |  |  |  |
| **Intercept** | -2.7138 | 0.7812 | 0.0005 | 0.066 |

SE=Standard Error; OR = Odds Ratio; Ref = Reference; F = Type III F-statistic; p = p-value; No. observations = 4836; No. individuals = 625; -2 Res Log Pseudo-Likelihood = 22,712.49; Pseudo-AIC = 22,714.49; Pseudo-BIC = 22,718.93. The difference of the study sample (n=683) to 625 subjects in the regression analysis is due to missing values in the independent and/or dependent variables.

## Table 7 - Results of the multilevel mixed effects logit regression analysis with the preference to seek medical services for the assessed case vignette as the dependent variable (625 subjects, yes=1, interaction age x gender x employment status)

| **Independent Variable** | **Coefficient** | **SE** | **p-value** | **OR** |
| --- | --- | --- | --- | --- |
| **Predisposing factors of participant** |  |  |  |  |
| Gender (male = 1) | 0.6990 | 0.8498 | 0.4108 | 2.012 |
| Age (metric in years) | -0.0159 | 0.0131 | 0.2245 | 0.984 |
| Educational level (CASMIN) (high = 1) | 0.2891 | 0.1440 | 0.0447 | 1.335 |
| **Enabling factors of participant** |  |  |  |  |
| Employment status (employed/in training/studying = 1) | -0.1797 | 0.9090 | 0.8433 | 0.836 |
| Regular doctor (yes = 1) | 0.8700 | 0.3286 | 0.0081 | 2.387 |
| **Need factor of participant** |  |  |  |  |
| European Index of the EQ-5D (metric in units) | 0.0027 | 0.0043 | 0.5355 | 1.003 |
| **Attributes of case vignette** |  |  |  |  |
| Mobility (problems = 1) | 0.6811 | 0.0778 | <0.0001 | 1.976 |
| Self-Care (problems = 1) | 1.0319 | 0.0982 | <0.0001 | 2.806 |
| Usual Activities (problems = 1) | 1.0352 | 0.0802 | <0.0001 | 2.816 |
| Pain/Discomfort (problems = 1) | 1.3819 | 0.0787 | <0.0001 | 3.983 |
| Anxiety/Depression (problems = 1) | 0.5154 | 0.0833 | <0.0001 | 1.674 |
| **Other factors** |  |  |  |  |
| Version of questionnaire (first questions on own health = 1) | 0.1387 | 0.1261 | 0.2715 | 1.149 |
| Certainty of choice (NRS 9-10 = 1) | -0.6213 | 0.1025 | <0.0001 | 0.537 |
| **Interactions** |  |  |  |  |
| Age x gender x employment status; F = 0.29, p = 0.8323 |  |  |  |  |
| Age x male x employment status = 1 | -0.0030 | 0.0206 | 0.8834 | 0.997 |
| Age x male x employment status = 0  Age x female x employment status = 1 | -0.0032  0.0051 | 0.0142  0.0162 | 0.8196  0.7521 | 0.997  1.005 |
| Age x female x employment status = 0 | Ref |  |  |  |
| **Intercept** | -2.7261 | 0.9188 | 0.0031 | 0.065 |

SE=Standard Error; OR = Odds Ratio; Ref = Reference; F = Type III F-statistic; p = p-value; No. observations = 4836; No. individuals = 625; -2 Res Log Pseudo-Likelihood = 22,712.96; Pseudo-AIC = 22,714.96; Pseudo-BIC = 22,719.39. The difference of the study sample (n=683) to 625 subjects in the regression analysis is due to missing values in the independent and/or dependent variables.

## Table 8 - Results of the multilevel mixed effects logit regression analysis with the preferred time until consultation for the assessed case vignette as the dependent variable (624 subjects, immediately, today or tomorrow=1, interaction age x gender)

| **Independent Variable** | **Coefficient** | **SE** | **p-value** | **OR** |
| --- | --- | --- | --- | --- |
| **Predisposing factors of participant** |  |  |  |  |
| Gender (male = 1) | -2.0524 | 1.2481 | 0.1002 | 0.128 |
| Age (metric in years) | -0.0213 | 0.0165 | 0.1969 | 0.979 |
| Educational level (CASMIN) (high = 1) | -0.6258 | 0.2546 | 0.0140 | 0.535 |
| **Enabling factors of participant** |  |  |  |  |
| Employment status (employed/in training/studying = 1) | 0.2326 | 0.2669 | 0.3836 | 1.262 |
| Regular doctor (yes = 1) | 0.5054 | 0.5926 | 0.3937 | 1.658 |
| **Need factor of participant** |  |  |  |  |
| European Index of the EQ-5D (metric in units) | 0.0016 | 0.0071 | 0.8275 | 1.002 |
| **Attributes of case vignette** |  |  |  |  |
| Mobility (problems = 1) | 0.4389 | 0.1317 | 0.0009 | 1.551 |
| Self-Care (problems = 1) | 1.0025 | 0.1611 | <0.0001 | 2.725 |
| Usual Activities (problems = 1) | 0.6927 | 0.1417 | <0.0001 | 1.999 |
| Pain/Discomfort (problems = 1) | 1.0896 | 0.1314 | <0.0001 | 2.973 |
| Anxiety/Depression (problems = 1) | 0.1528 | 0.1456 | 0.2941 | 1.165 |
| **Other factors** |  |  |  |  |
| Version of questionnaire (first questions on own health = 1) | 0.8026 | 0.2132 | 0.0002 | 2.231 |
| Certainty of choice (NRS 9-10 = 1) | 0.8286 | 0.1773 | <0.0001 | 2.290 |
| **Interactions** |  |  |  |  |
| Age x gender; F = 4.42, p = 0.0356 |  |  |  |  |
| Age x male | 0.0477 | 0.0227 | 0.0356 | 1.049 |
| Age x female | Ref |  |  |  |
| **Intercept** | -4.9263 | 1.2944 | 0.0002 | 0.007 |

SE=Standard Error; OR = Odds Ratio; Ref = Reference; F = Type III F-statistic; p = p-value; No. observations = 4786; No. individuals = 624; -2 Res Log Pseudo-Likelihood = 27,921.94; Pseudo-AIC = 27,923.94; Pseudo-BIC = 27,928.37. The difference of the study sample (n=683) to 624 subjects in the regression analysis is due to missing values in the independent and/or dependent variables.

## Table 9 - Results of the multilevel mixed effects logit regression analysis with the preferred time until consultation for the assessed case vignette as the dependent variable (624 subjects, immediately, today or tomorrow=1, interaction age x gender x educational level)

| **Independent Variable** | **Coefficient** | **SE/^†^F-Statistic** | **p-value** | **OR** |
| --- | --- | --- | --- | --- |
| **Predisposing factors of participant** |  |  |  |  |
| Gender (male = 1) | -2.1045 | 1.2520 | 0.0928 | 0.122 |
| Age (metric in years) | -0.0191 | 0.0179 | 0.2848 | 0.981 |
| Educational level (CASMIN) (high = 1) | 0.0876 | 1.4296 | 0.9511 | 1.092 |
| **Enabling factors of participant** |  |  |  |  |
| Employment status (employed/in training/studying = 1) | 0.2443 | 0.2679 | 0.3619 | 1.277 |
| Regular doctor (yes = 1) | 0.4338 | 0.6004 | 0.4700 | 1.543 |
| **Need factor of participant** |  |  |  |  |
| European Index of the EQ-5D (metric in units) | 0.0013 | 0.0071 | 0.8575 | 1.001 |
| **Attributes of case vignette** |  |  |  |  |
| Mobility (problems = 1) | 0.4449 | 0.1320 | 0.0008 | 1.560 |
| Self-Care (problems = 1) | 1.0079 | 0.1613 | <0.0001 | 2.740 |
| Usual Activities (problems = 1) | 0.6988 | 0.1420 | <0.0001 | 2.011 |
| Pain/Discomfort (problems = 1) | 1.0943 | 0.1318 | <0.0001 | 2.987 |
| Anxiety/Depression (problems = 1) | 0.1524 | 0.1458 | 0.2961 | 1.165 |
| **Other factors** |  |  |  |  |
| Version of questionnaire (first questions on own health = 1) | 0.8292 | 0.2140 | 0.0001 | 2.291 |
| Certainty of choice (NRS 9-10 = 1) | 0.8411 | 0.1777 | <0.0001 | 2.319 |
| **Interactions** |  |  |  |  |
| Age x gender x educational level (CASMIN); F = 3.42, p = 0.0165 |  |  |  |  |
| Age x male x CASMIN = 3  Age x male x CASMIN = 1-2  Age x female x CASMIN = 3  Age x female x CASMIN = 1-2 | 0.0309  0.0538  -0.0023  Ref | 0.0349  0.0229  0.0268 | 0.3765  0.0189  0.9328 | 1.031  1.055  0.998 |
| **Intercept** | -5.1278 | 1.3519 | 0.0002 | 0.006 |

SE=Standard Error; OR = Odds Ratio; Ref = Reference; F = Type III F-statistic; p = p-value; No. observations = 4786; No. individuals = 624; -2 Res Log Pseudo-Likelihood = 27,991.06; Pseudo-AIC = 27,993.06; Pseudo-BIC = 27,997.50. The difference of the study sample (n=683) to 624 subjects in the regression analysis is due to missing values in the independent and/or dependent variables.

## Table 10 - Results of the multilevel mixed effects logit regression analysis with the preferred time until consultation for the assessed case vignette as the dependent variable (624 subjects, immediately, today or tomorrow=1, interaction age x gender x employment status)

| **Independent Variable** | **Coefficient** | **SE** | **p-value** | **OR** |
| --- | --- | --- | --- | --- |
| **Predisposing factors of participant** |  |  |  |  |
| Gender (male = 1) | -1.7432 | 1.4657 | 0.2344 | 0.175 |
| Age (metric in years) | -0.0115 | 0.0232 | 0.6210 | 0.989 |
| Educational level (CASMIN) (high = 1) | -0.6257 | 0.2554 | 0.0143 | 0.535 |
| **Enabling factors of participant** |  |  |  |  |
| Employment status (employed/in training/studying = 1) | 1.0268 | 1.5732 | 0.5140 | 2.792 |
| Regular doctor (yes = 1) | 0.5125 | 0.5964 | 0.3902 | 1.669 |
| **Need factor of participant** |  |  |  |  |
| European Index of the EQ-5D (metric in units) | 0.0011 | 0.0072 | 0.8754 | 1.001 |
| **Attributes of case vignette** |  |  |  |  |
| Mobility (problems = 1) | 0.4404 | 0.1318 | 0.0008 | 1.553 |
| Self-Care (problems = 1) | 1.0036 | 0.1612 | <0.0001 | 2.728 |
| Usual Activities (problems = 1) | 0.6947 | 0.1418 | <0.0001 | 2.003 |
| Pain/Discomfort (problems = 1) | 1.0915 | 0.1316 | <0.0001 | 2.979 |
| Anxiety/Depression (problems = 1) | 0.1517 | 0.1457 | 0.2981 | 1.164 |
| **Other factors** |  |  |  |  |
| Version of questionnaire (first questions on own health = 1) | 0.8101 | 0.2142 | 0.0002 | 2.248 |
| Certainty of choice (NRS 9-10 = 1) | 0.8351 | 0.1783 | <0.0001 | 2.305 |
| **Interactions** |  |  |  |  |
| Age x gender x employment status; F = 1.64, p = 0.1770 |  |  |  |  |
| Age x male x employment status = 1 | 0.0283 | 0.3672 | 0.4411 | 1.029 |
| Age x male x employment status = 0  Age x female x employment status = 1 | 0.0443  -0.0120 | 0.0244  0.0280 | 0.0698  0.6679 | 1.045  0.988 |
| Age x female x employment status = 0 | Ref |  |  |  |
| **Intercept** | -5.5591 | 1.6251 | 0.0007 | 0.004 |

SE=Standard Error; OR = Odds Ratio; Ref = Reference; F = Type III F-statistic; p = p-value; No. observations = 4786; No. individuals = 624; -2 Res Log Pseudo-Likelihood = 27,962.61; Pseudo-AIC = 27,964.61; Pseudo-BIC = 27,969.04. The difference of the study sample (n=683) to 624 subjects in the regression analysis is due to missing values in the independent and/or dependent variables.

## Table 11 - Results of the multilevel mixed effects logit regression analysis with the preferred time until consultation for the assessed case vignette as the dependent variable (301 subjects, immediately, today or tomorrow=1, version of questionnaire 1)

| **Independent Variable** | **Coefficient** | **SE** | **p-value** | **OR** |
| --- | --- | --- | --- | --- |
| **Predisposing factors of participant** |  |  |  |  |
| Gender (male = 1) | 0.7974 | 0.2971 | 0.0073 | 2.220 |
| Age (metric in years) | -0.0041 | 0.0181 | 0.8226 | 0.996 |
| Educational level (CASMIN) (high = 1) | -0.5324 | 0.3416 | 0.1193 | 0.587 |
| **Enabling factors of participant** |  |  |  |  |
| Employment status (employed/in training/studying = 1) | 0.1285 | 0.3676 | 0.7266 | 1.137 |
| **Need factor of participant** |  |  |  |  |
| European Index of the EQ-5D (metric in units) | 0.0139 | 0.0109 | 0.2010 | 1.014 |
| **Attributes of case vignette** |  |  |  |  |
| Mobility (problems = 1) | 0.6818 | 0.1735 | <0.0001 | 1.977 |
| Self-Care (problems = 1) | 0.9387 | 0.2136 | <0.0001 | 2.557 |
| Usual Activities (problems = 1) | 0.7708 | 0.1850 | <0.0001 | 2.161 |
| Pain/Discomfort (problems = 1) | 1.1848 | 0.1725 | <0.0001 | 3.270 |
| Anxiety/Depression (problems = 1) | 0.2328 | 0.1905 | 0.2219 | 1.262 |
| **Other factors** |  |  |  |  |
| Certainty of choice (NRS 9-10 = 1) | 0.7613 | 0.2317 | 0.0010 | 2.141 |
| **Intercept** | -5.8813 | 1.3753 | <0.0001 | 0.003 |

SE=Standard Error; OR = Odds Ratio; No. observations = 2300; No. individuals = 301; -2 Res Log Pseudo-Likelihood = 12,972.00; Pseudo-AIC = 12,974.00; Pseudo-BIC = 12,977.71.

## Table 12 - Results of the multilevel mixed effects logit regression analysis with the preferred time until consultation for the assessed case vignette as the dependent variable (323 subjects, immediately, today or tomorrow=1, version of questionnaire 2)

| **Independent Variable** | **Coefficient** | **SE** | **p-value** | **OR** |
| --- | --- | --- | --- | --- |
| **Predisposing factors of participant** |  |  |  |  |
| Gender (male = 1) | 0.2144 | 0.3132 | 0.4938 | 1.239 |
| Age (metric in years) | 0.0038 | 0.0199 | 0.8497 | 1.004 |
| Educational level (CASMIN) (high = 1) | -0.8351 | 0.3890 | 0.0319 | 0.434 |
| **.Enabling factors of participant** |  |  |  |  |
| Employment status (employed/in training/studying = 1) | 0.2189 | 0.3981 | 0.5825 | 1.245 |
| **Need factor of participant** |  |  |  |  |
| European Index of the EQ-5D (metric in units) | -0.0054 | 0.0095 | 0.5681 | 0.995 |
| **Attributes of case vignette** |  |  |  |  |
| Mobility (problems = 1) | 0.1316 | 0.2069 | 0.5247 | 1.141 |
| Self-Care (problems = 1) | 1.1378 | 0.2478 | <0.0001 | 3.120 |
| Usual Activities (problems = 1) | 0.6300 | 0.2236 | 0.0049 | 1.878 |
| Pain/Discomfort (problems = 1) | 0.9855 | 0.2061 | <0.0001 | 2.679 |
| Anxiety/Depression (problems = 1) | 0.0713 | 0.2284 | 0.7551 | 1.074 |
| **Other factors** |  |  |  |  |
| Certainty of choice (NRS 9-10 = 1) | 0.8514 | 0.2797 | 0.0024 | 2.343 |
| **Intercept** | -4.7764 | 1.5122 | 0.0017 | 0.008 |

SE=Standard Error; OR = Odds Ratio; No. observations = 2486; No. individuals = 323; -2 Res Log Pseudo-Likelihood = 14,959.69; Pseudo-AIC = 14,961.69; Pseudo-BIC = 14,965.47.
